# Crystal structure of (3S*,4S*,4aS*,5R*,6R*,6aS*,7R*,11aS*,11bR*)-5,6-bis(benzo­yloxy)-3,4a-dihy­droxy-4,7,11b-trimethyl-1,2,3,4,4a,5,6,6a,7,11,11a,11b-dodeca­hydro­phenanthro[3,2-*b*]furan-4-carb­oxy­lic acid methanol monosolvate

**DOI:** 10.1107/S2056989015016461

**Published:** 2015-09-12

**Authors:** Sadaf Siddiqui, Osayemwenre Erharuyi, Abiodun Falodun, M. Iqbal Choudhary, Sammer Yousuf

**Affiliations:** a H.E.J. Research Institute of Chemistry, International Center for Chemical and Biological Sciences, University of Karachi, Karachi 75270, Pakistan; b Department of Pharmaceutical Chemistry, Faculty of Pharmacy, University of Benin, Benin City, Nigeria; c Department of Biochemistry, Faculty of Science, King Abdul Aziz University, Jeddah-21412, Saudi Arabia

**Keywords:** crystal structure, diterpenoid, *Caesalpinia pulcherrima*, Pulcherrimin A, hydrogen bonding

## Abstract

The title compound, C_34_H_36_O_9_·CH_3_OH, is a diterpenoid isolated from the roots of *Caesalpinia pulcherrima* (L.) Swartz. The three *trans*-fused six-membered rings are in chair, chair and half-chair conformations. The mean plane of this fused-ring system makes dihedral angles of 67.95 (15) and 83.72 (14)° with the two phenyl rings of the benzo­yloxy groups. An intra­molecular C—H⋯O hydrogen bond is observed. In the crystal, mol­ecules are linked *via* O—H⋯O hydrogen bonds, forming an infinite chain along the *b-*axis direction.

## Related literature   

For background to *Caesalpinia pulcherrima* (L.) Swartz and its biological activities, see: Pawar *et al.* (2009 ▸); Udenigwe *et al.* (2007 ▸); Sudhakar *et al.* (2006 ▸); Gupta *et al.* (2000 ▸); Patil *et al.* (1997 ▸). For the biological applications of Pulcherrimin A, see: Yodsaoue *et al.* (2011 ▸); Patil *et al.* (1997 ▸). For the crystal structure of a related compound, see: Fun *et al.* (2010 ▸).
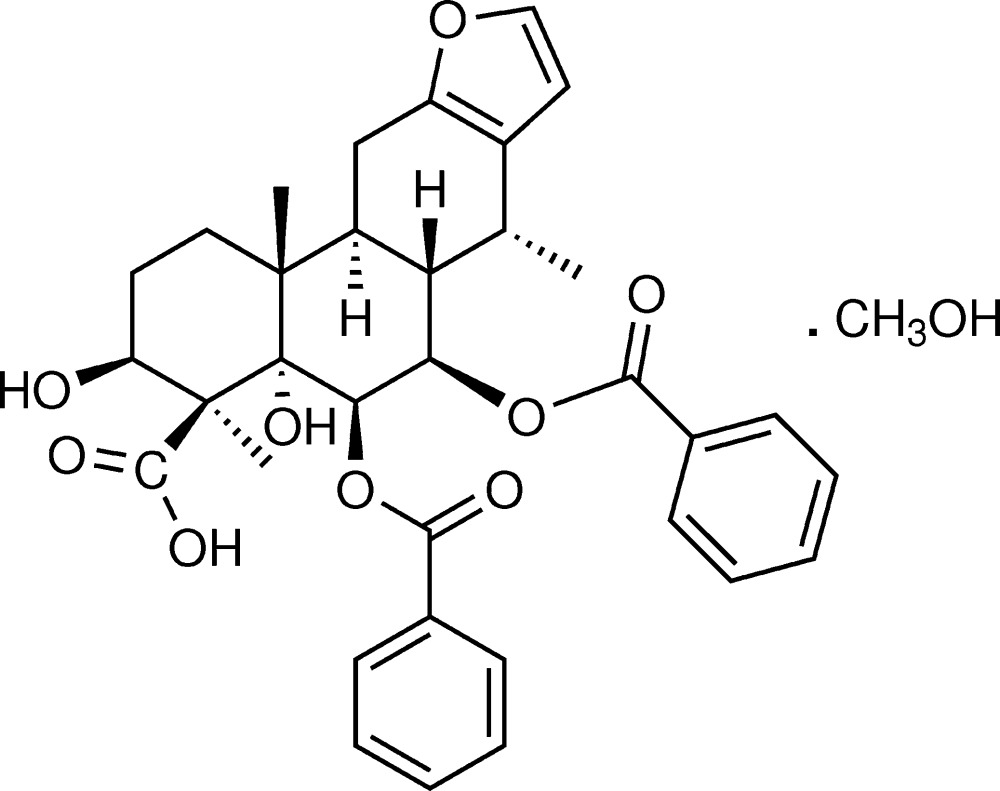


## Experimental   

### Crystal data  

C_34_H_36_O_9_·CH_4_O
*M* *_r_* = 620.67
Orthorhombic, 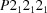

*a* = 11.7943 (6) Å
*b* = 13.5934 (7) Å
*c* = 19.2988 (11) Å
*V* = 3094.1 (3) Å^3^
*Z* = 4
Mo *K*α radiation
μ = 0.10 mm^−1^
*T* = 293 K
0.45 × 0.30 × 0.10 mm

### Data collection  

Bruker SMART APEX CCD area-detector diffractometer
18238 measured reflections
3916 independent reflections
2882 reflections with *I* > 2σ(*I*)
*R*_int_ = 0.049

### Refinement  

*R*[*F* ^2^ > 2σ(*F* ^2^)] = 0.052
*wR*(*F* ^2^) = 0.139
*S* = 1.03
3916 reflections
411 parameters
13 restraints
H-atom parameters constrained
Δρ_max_ = 0.30 e Å^−3^
Δρ_min_ = −0.18 e Å^−3^

### 

Data collection: *SMART* (Bruker, 2000 ▸); cell refinement: *SAINT* (Bruker, 2000 ▸); data reduction: *SAINT*; program(s) used to solve structure: *SHELXS97* (Sheldrick, 2008 ▸); program(s) used to refine structure: *SHELXL97* (Sheldrick, 2008 ▸); molecular graphics: *SHELXTL* (Sheldrick, 2008 ▸); software used to prepare material for publication: *SHELXTL* and *PLATON* (Spek, 2009 ▸).

## Supplementary Material

Crystal structure: contains datablock(s) global, I. DOI: 10.1107/S2056989015016461/is5414sup1.cif

Structure factors: contains datablock(s) I. DOI: 10.1107/S2056989015016461/is5414Isup2.hkl

Supporting information file. DOI: 10.1107/S2056989015016461/is5414Isup3.cml

**Fig. 1.**
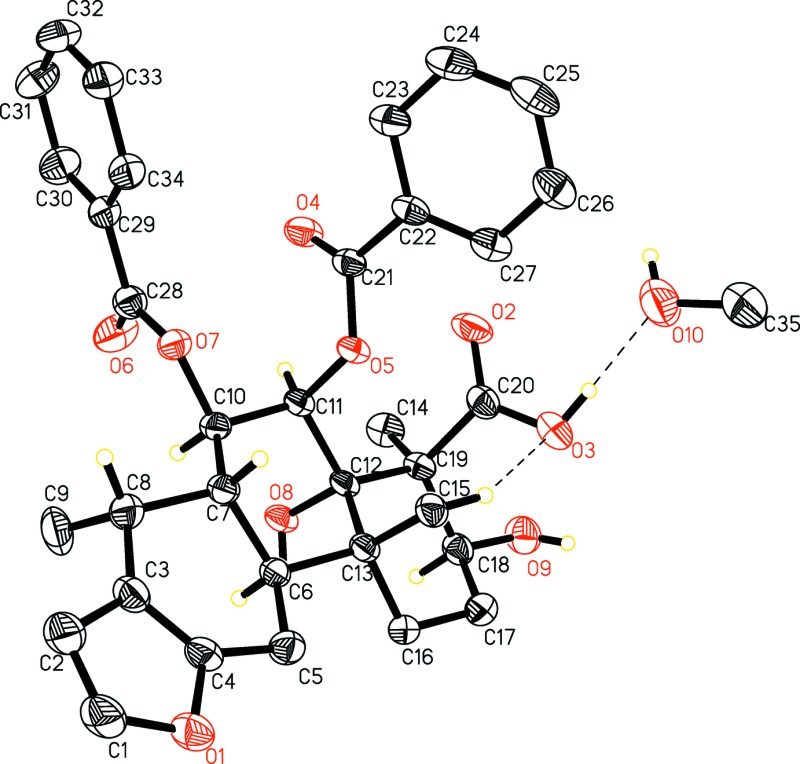
The molecular structure of the title compound with displacement ellipsoids drawn at the 30% probability level. Only H atoms related to stereochemistry, of OH groups and involved in the hydrogen bonds are shown.

**Fig. 2.**
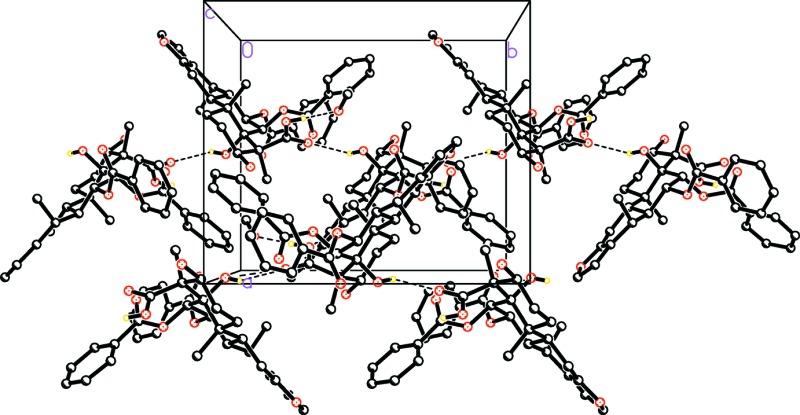
A packing diagram of the title compound viewed perpendicular to the ab plane. Only H atoms involved in the O—H···O hydrogen bonds are shown.

. DOI: 10.1107/S2056989015016461/is5414fig1.tif
The mol­ecular structure of the title compound with displacement ellipsoids drawn at the 30% probability level. Only H atoms related to stereochemistry, of OH groups and involved in the hydrogen bonds are shown.

ab . DOI: 10.1107/S2056989015016461/is5414fig2.tif
A packing diagram of the title compound viewed perpendicular to the *ab* plane. Only H atoms involved in the O—H⋯O hydrogen bonds are shown.

CCDC reference: 1422031

Additional supporting information:  crystallographic information; 3D view; checkCIF report

## Figures and Tables

**Table 1:** Hydrogen-bond geometry (, )

*D*H*A*	*D*H	H*A*	*D* *A*	*D*H*A*
O3H3O10	0.84	1.79	2.626(5)	177
O8H8O2^i^	0.84	2.03	2.728(3)	141
O10H10O8^ii^	0.84	2.26	2.967(4)	142
C15H15*A*O3	0.96	2.35	3.231(5)	152

Symmetry codes: (i) 
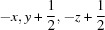
; (ii) 
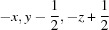
.

## supplementary crystallographic information

### S1. Comment

*Caesalpinia pulcherrima* (*L*.) Swartz, commonly called peacock flower, belongs to the Caesalpiniaceae family (Patil *et al.*, 1997). Pharmacological study of the plant reveals anti-microbial (Sudhakar *et al.*, 2006), antioxidant (Pawar *et al.*, 2009), antidiabetic, anticancer, antirheumatic (Udenigwe *et al.*, 2007) and anti-tumor (Gupta *et al.*, 2000) properties. The title compound, also called pulcherrimin A, was previously isolated by Patil and co-workers (1997) from the roots of *Caesalpinia pulcherrima*. The compound is known to have anti-inflammatory (Yodsaoue *et al.*, 2011) and discriminating effect on DNA-deficient yeast mutants (Patil *et al.*, 1997). Herein we report the isolation and single-crystal X-ray diffraction studies of pulcherrimin A methanol solvate. The structure of the title compound is similar to that of previously published isovouacapenol C (Fun *et al.*, 2010) with the difference that hydroxyl and methyl groups attached at C-24 and C-11 were substituted by benzoyl (O4/O10/C25–C31) and carboxylic acid (O5/O6/C35) groups, respectively. In addition, an equatorially oriented hydroxyl group was found to be attached at C-12. All bond lengths and angles were found to be similar to that of related structure (Fun *et al.*, 2010). In the crystal packing of the title compound, molecules are linked *via* O8—H8—O2 and O10—H10—O8 hydrogen bonds (Fig. 2 and Table 1) that forms a chain structure running along the *b* axis.

### S2. Experimental

Powdered *Caesalpinia pulcherrima* (*L*.) Swartz roots (2.9 kg) were soaked in methanol (7.5 *L*) at room temperature. After 7 days methanolic extract was filtered and concentrated to obtain a crude gummy material (240 g). The concentrated extract was suspended in water and partitioned into petroleum ether, chloroform and ethyl acetate soluble parts by solvent-solvent extraction. The dried chloroform extract was fractionated into six fractions (F1—F6) by column chromatography over silica gel. Fraction F6 was re-chromatographed on silica gel, eluting with dichloromethane increasing polarity with methanol. Recrystallization of the crystalline solid obtained in methanol yielded 177.6 mg of title compound.

### S3. Refinement

H atoms on methyl, phenyl, methine, methylene and oxygen were positioned geometrically with C—H = 0.96 Å (CH_3_), 0.93 Å (CH phenyl), 0.98 Å (CH), 0.97 Å (CH_2_) and 0.84 Å (OH) and constrained to ride on their parent atoms with *U*_iso_(H) = 1.2*U*_eq_(CH and CH_2_), 1.5 *U*_eq_(CH_3_) and 1.2–1.5*U*_eq_(OH). Restraints on a bond length [C—O = 1.50 (1) Å] and displacement parameters (*ISOR*) were applied for atoms C35 and O10. In the absence of significant anomalous scattering effects, Friedel pairs have been merged.

### Crystal data

**Table:**

C_34_H_36_O_9_·CH_4_O	*F*(000) = 1320
*M**_r_* = 620.67	*D*_x_ = 1.332 Mg m^−^^3^
Orthorhombic, *P*2_1_2_1_2_1_	Mo *K*α radiation, λ = 0.71073 Å
Hall symbol: P 2ac 2ab	Cell parameters from 2827 reflections
*a* = 11.7943 (6) Å	θ = 2.3–24.7°
*b* = 13.5934 (7) Å	µ = 0.10 mm^−^^1^
*c* = 19.2988 (11) Å	*T* = 293 K
*V* = 3094.1 (3) Å^3^	Prism, colorless
*Z* = 4	0.45 × 0.30 × 0.10 mm

### Data collection

**Table:**

Bruker SMART APEX CCD area-detector diffractometer	2882 reflections with *I* > 2σ(*I*)
Radiation source: fine-focus sealed tube	*R*_int_ = 0.049
Graphite monochromator	θ_max_ = 27.5°, θ_min_ = 1.8°
ω scan	*h* = −14→14
18238 measured reflections	*k* = −7→17
3916 independent reflections	*l* = −25→25

### Refinement

**Table:**

Refinement on *F*^2^	Primary atom site location: structure-invariant direct methods
Least-squares matrix: full	Secondary atom site location: difference Fourier map
*R*[*F*^2^ > 2σ(*F*^2^)] = 0.052	Hydrogen site location: inferred from neighbouring sites
*wR*(*F*^2^) = 0.139	H-atom parameters constrained
*S* = 1.03	*w* = 1/[σ^2^(*F*_o_^2^) + (0.0769*P*)^2^ + 0.1739*P*] where *P* = (*F*_o_^2^ + 2*F*_c_^2^)/3
3916 reflections	(Δ/σ)_max_ = 0.001
411 parameters	Δρ_max_ = 0.30 e Å^−^^3^
13 restraints	Δρ_min_ = −0.18 e Å^−^^3^

### Fractional atomic coordinates and isotropic or equivalent isotropic displacement parameters (Å^2^)

**Table:**

	*x*	*y*	*z*	*U*_iso_*/*U*_eq_	
O1	0.4694 (2)	1.25976 (18)	0.17389 (15)	0.0559 (7)	
O2	0.0387 (3)	0.72827 (18)	0.27984 (16)	0.0644 (8)	
O3	0.1186 (3)	0.7991 (2)	0.36988 (16)	0.0703 (9)	
H3	0.1319	0.7403	0.3803	0.084*	
O4	0.0845 (2)	0.72060 (18)	0.11387 (14)	0.0523 (7)	
O5	0.18140 (19)	0.82310 (14)	0.18442 (11)	0.0379 (5)	
O6	−0.0009 (2)	0.9189 (2)	0.01641 (15)	0.0697 (9)	
O7	0.1773 (2)	0.90679 (17)	0.05536 (11)	0.0417 (5)	
O8	−0.0053 (2)	1.03030 (16)	0.20910 (12)	0.0425 (6)	
H8	0.0011	1.0840	0.2303	0.064*	
O9	−0.0646 (3)	0.9195 (2)	0.41210 (14)	0.0669 (8)	
H9	−0.0195	0.9114	0.4453	0.100*	
O10	0.1596 (4)	0.6133 (3)	0.3973 (2)	0.1137 (15)	
H10	0.1259	0.5658	0.3785	0.171*	
C1	0.5090 (4)	1.2791 (3)	0.1087 (2)	0.0647 (12)	
H1	0.5657	1.3245	0.0988	0.078*	
C2	0.4571 (4)	1.2255 (3)	0.0619 (2)	0.0627 (11)	
H2	0.4702	1.2262	0.0144	0.075*	
C3	0.3767 (3)	1.1659 (3)	0.09769 (19)	0.0484 (9)	
C4	0.3866 (3)	1.1895 (3)	0.1646 (2)	0.0462 (8)	
C5	0.3261 (3)	1.1496 (3)	0.22463 (19)	0.0472 (9)	
H5A	0.2985	1.2033	0.2531	0.057*	
H5B	0.3778	1.1105	0.2524	0.057*	
C6	0.2257 (3)	1.0853 (2)	0.20139 (16)	0.0365 (7)	
H6	0.1631	1.1302	0.1904	0.044*	
C7	0.2520 (3)	1.0271 (2)	0.13434 (16)	0.0388 (7)	
H7	0.3137	0.9810	0.1443	0.047*	
C8	0.2893 (3)	1.0936 (3)	0.07297 (18)	0.0500 (9)	
H8A	0.3258	1.0512	0.0385	0.060*	
C9	0.1936 (4)	1.1480 (3)	0.0365 (2)	0.0759 (14)	
H9A	0.2241	1.1860	−0.0010	0.114*	
H9B	0.1566	1.1910	0.0689	0.114*	
H9C	0.1399	1.1013	0.0188	0.114*	
C10	0.1479 (3)	0.9680 (2)	0.11460 (16)	0.0384 (7)	
H10A	0.0879	1.0137	0.1006	0.046*	
C11	0.1013 (3)	0.9004 (2)	0.17067 (16)	0.0359 (7)	
H11	0.0305	0.8710	0.1540	0.043*	
C12	0.0757 (3)	0.9609 (2)	0.23601 (17)	0.0357 (7)	
C13	0.1838 (3)	1.0178 (2)	0.26167 (17)	0.0360 (7)	
C14	−0.1111 (3)	0.8765 (3)	0.2674 (2)	0.0551 (10)	
H14A	−0.1523	0.8416	0.3025	0.083*	
H14B	−0.1047	0.8360	0.2268	0.083*	
H14C	−0.1507	0.9360	0.2559	0.083*	
C15	0.2801 (3)	0.9489 (3)	0.28529 (19)	0.0442 (8)	
H15A	0.2532	0.9072	0.3220	0.066*	
H15B	0.3428	0.9875	0.3017	0.066*	
H15C	0.3042	0.9091	0.2469	0.066*	
C16	0.1502 (3)	1.0825 (2)	0.32402 (16)	0.0427 (8)	
H16A	0.2171	1.1157	0.3415	0.051*	
H16B	0.0971	1.1325	0.3087	0.051*	
C17	0.0964 (4)	1.0236 (3)	0.38239 (19)	0.0518 (9)	
H17A	0.1506	0.9759	0.3998	0.062*	
H17B	0.0763	1.0674	0.4201	0.062*	
C18	−0.0080 (3)	0.9710 (3)	0.35714 (18)	0.0476 (9)	
H18	−0.0606	1.0225	0.3419	0.057*	
C19	0.0082 (3)	0.9019 (2)	0.29442 (17)	0.0408 (8)	
C20	0.0577 (3)	0.8022 (3)	0.3134 (2)	0.0483 (9)	
C21	0.1623 (3)	0.7356 (2)	0.15241 (18)	0.0398 (8)	
C22	0.2491 (3)	0.6604 (2)	0.17071 (19)	0.0418 (8)	
C23	0.2675 (3)	0.5849 (3)	0.1240 (2)	0.0512 (9)	
H23	0.2303	0.5844	0.0815	0.061*	
C24	0.3419 (4)	0.5101 (3)	0.1413 (3)	0.0645 (12)	
H24	0.3564	0.4601	0.1096	0.077*	
C25	0.3941 (3)	0.5091 (3)	0.2045 (3)	0.0631 (12)	
H25	0.4421	0.4574	0.2161	0.076*	
C26	0.3761 (3)	0.5835 (3)	0.2506 (2)	0.0614 (11)	
H26	0.4118	0.5823	0.2936	0.074*	
C27	0.3049 (3)	0.6606 (3)	0.2337 (2)	0.0522 (9)	
H27	0.2946	0.7124	0.2645	0.063*	
C28	0.0917 (3)	0.8830 (3)	0.01251 (19)	0.0468 (9)	
C29	0.1236 (3)	0.8057 (3)	−0.03808 (17)	0.0450 (8)	
C30	0.0424 (4)	0.7756 (3)	−0.0852 (2)	0.0634 (11)	
H30	−0.0287	0.8053	−0.0856	0.076*	
C31	0.0671 (5)	0.7011 (4)	−0.1319 (2)	0.0719 (13)	
H31	0.0128	0.6809	−0.1637	0.086*	
C32	0.1714 (5)	0.6575 (3)	−0.1309 (2)	0.0728 (13)	
H32	0.1872	0.6067	−0.1618	0.087*	
C33	0.2524 (4)	0.6874 (3)	−0.0856 (2)	0.0621 (11)	
H33	0.3235	0.6579	−0.0860	0.075*	
C34	0.2286 (4)	0.7623 (3)	−0.03847 (19)	0.0515 (9)	
H34	0.2839	0.7828	−0.0073	0.062*	
C35	0.2242 (6)	0.5792 (4)	0.4516 (3)	0.1029 (19)	
H35A	0.2113	0.6195	0.4917	0.154*	
H35B	0.3030	0.5818	0.4392	0.154*	
H35C	0.2034	0.5125	0.4619	0.154*	

### Atomic displacement parameters (Å^2^)

**Table:**

	*U*^11^	*U*^22^	*U*^33^	*U*^12^	*U*^13^	*U*^23^
O1	0.0554 (16)	0.0453 (14)	0.0669 (17)	−0.0164 (13)	0.0011 (14)	0.0026 (13)
O2	0.084 (2)	0.0307 (12)	0.0785 (19)	−0.0077 (14)	0.0104 (17)	−0.0003 (14)
O3	0.090 (2)	0.0456 (15)	0.0753 (19)	0.0088 (17)	−0.0095 (19)	0.0116 (15)
O4	0.0555 (15)	0.0359 (12)	0.0656 (16)	−0.0013 (13)	−0.0091 (14)	−0.0074 (12)
O5	0.0385 (12)	0.0261 (10)	0.0492 (12)	0.0024 (10)	−0.0027 (11)	−0.0016 (10)
O6	0.0553 (17)	0.084 (2)	0.0696 (17)	0.0195 (17)	−0.0182 (15)	−0.0230 (17)
O7	0.0440 (13)	0.0402 (12)	0.0410 (12)	0.0004 (11)	−0.0022 (11)	−0.0055 (11)
O8	0.0436 (13)	0.0292 (10)	0.0548 (14)	0.0070 (11)	−0.0053 (12)	−0.0012 (11)
O9	0.081 (2)	0.0635 (17)	0.0566 (16)	−0.0089 (18)	0.0196 (15)	0.0086 (15)
O10	0.147 (4)	0.074 (2)	0.120 (3)	−0.002 (3)	−0.062 (3)	0.018 (2)
C1	0.068 (3)	0.054 (2)	0.072 (3)	−0.023 (2)	0.014 (2)	0.006 (2)
C2	0.075 (3)	0.056 (2)	0.057 (2)	−0.018 (2)	0.018 (2)	0.004 (2)
C3	0.054 (2)	0.0383 (18)	0.053 (2)	−0.0049 (17)	0.0055 (19)	0.0056 (17)
C4	0.046 (2)	0.0353 (16)	0.058 (2)	−0.0065 (16)	−0.0005 (18)	0.0031 (17)
C5	0.049 (2)	0.0464 (18)	0.0461 (18)	−0.0091 (18)	−0.0019 (18)	−0.0021 (16)
C6	0.0391 (18)	0.0305 (15)	0.0399 (16)	−0.0005 (14)	−0.0025 (14)	0.0011 (14)
C7	0.0417 (18)	0.0329 (16)	0.0417 (16)	0.0000 (16)	−0.0030 (15)	−0.0014 (14)
C8	0.060 (2)	0.048 (2)	0.0418 (18)	−0.0085 (19)	0.0044 (17)	−0.0004 (17)
C9	0.093 (3)	0.070 (3)	0.065 (3)	−0.022 (3)	−0.025 (3)	0.032 (2)
C10	0.0430 (19)	0.0321 (15)	0.0400 (16)	0.0040 (15)	−0.0039 (15)	−0.0040 (14)
C11	0.0362 (17)	0.0284 (14)	0.0431 (16)	0.0024 (14)	−0.0018 (15)	−0.0003 (14)
C12	0.0396 (18)	0.0239 (13)	0.0434 (17)	0.0046 (13)	−0.0014 (15)	0.0018 (14)
C13	0.0382 (17)	0.0290 (14)	0.0408 (16)	0.0014 (14)	−0.0027 (15)	0.0016 (14)
C14	0.044 (2)	0.055 (2)	0.067 (2)	−0.0051 (19)	0.009 (2)	0.000 (2)
C15	0.045 (2)	0.0386 (17)	0.0490 (18)	0.0030 (16)	−0.0116 (17)	0.0031 (15)
C16	0.053 (2)	0.0359 (16)	0.0389 (16)	−0.0019 (16)	−0.0009 (16)	−0.0022 (15)
C17	0.065 (2)	0.048 (2)	0.0420 (18)	−0.001 (2)	0.0019 (19)	−0.0015 (17)
C18	0.056 (2)	0.0403 (18)	0.0460 (19)	0.0056 (18)	0.0126 (18)	0.0049 (16)
C19	0.0431 (19)	0.0310 (15)	0.0484 (18)	−0.0010 (16)	0.0049 (16)	0.0001 (15)
C20	0.050 (2)	0.0416 (19)	0.053 (2)	−0.0004 (17)	0.0092 (19)	0.0083 (18)
C21	0.044 (2)	0.0291 (15)	0.0461 (18)	−0.0032 (15)	0.0049 (17)	−0.0020 (14)
C22	0.0390 (18)	0.0289 (15)	0.058 (2)	−0.0018 (15)	0.0099 (17)	−0.0001 (15)
C23	0.054 (2)	0.0381 (18)	0.062 (2)	0.0010 (18)	0.011 (2)	−0.0062 (18)
C24	0.058 (3)	0.044 (2)	0.092 (3)	0.010 (2)	0.017 (3)	−0.011 (2)
C25	0.043 (2)	0.0403 (19)	0.106 (4)	0.0108 (18)	0.009 (2)	0.007 (2)
C26	0.049 (2)	0.050 (2)	0.085 (3)	0.0040 (19)	−0.007 (2)	0.007 (2)
C27	0.050 (2)	0.0388 (18)	0.067 (2)	0.0048 (18)	0.001 (2)	−0.0040 (18)
C28	0.047 (2)	0.0454 (19)	0.0482 (19)	0.0047 (18)	−0.0029 (18)	−0.0011 (17)
C29	0.054 (2)	0.0417 (18)	0.0396 (17)	−0.0030 (17)	0.0006 (17)	−0.0022 (16)
C30	0.058 (3)	0.068 (3)	0.064 (2)	−0.005 (2)	−0.007 (2)	−0.010 (2)
C31	0.081 (3)	0.078 (3)	0.057 (2)	−0.009 (3)	−0.013 (2)	−0.022 (2)
C32	0.100 (4)	0.056 (2)	0.062 (3)	0.001 (3)	0.012 (3)	−0.021 (2)
C33	0.073 (3)	0.056 (2)	0.057 (2)	0.007 (2)	0.009 (2)	−0.005 (2)
C34	0.057 (2)	0.049 (2)	0.0490 (19)	−0.0029 (19)	−0.0032 (19)	−0.0026 (17)
C35	0.131 (5)	0.071 (3)	0.106 (4)	0.001 (3)	−0.021 (4)	0.009 (3)

### Geometric parameters (Å, º)

**Table:**

O1—C1	1.367 (5)	C13—C16	1.542 (4)
O1—C4	1.377 (4)	C14—C19	1.540 (5)
O2—C20	1.216 (5)	C14—H14A	0.9600
O3—C20	1.306 (5)	C14—H14B	0.9600
O3—H3	0.8400	C14—H14C	0.9600
O4—C21	1.198 (4)	C15—H15A	0.9600
O5—C21	1.359 (4)	C15—H15B	0.9600
O5—C11	1.438 (4)	C15—H15C	0.9600
O6—C28	1.199 (4)	C16—C17	1.521 (5)
O7—C28	1.345 (4)	C16—H16A	0.9700
O7—C10	1.456 (4)	C16—H16B	0.9700
O8—C12	1.439 (4)	C17—C18	1.505 (6)
O8—H8	0.8400	C17—H17A	0.9700
O9—C18	1.435 (4)	C17—H17B	0.9700
O9—H9	0.8400	C18—C19	1.544 (5)
O10—C35	1.376 (6)	C18—H18	0.9800
O10—H10	0.8400	C19—C20	1.521 (5)
C1—C2	1.313 (6)	C21—C22	1.489 (5)
C1—H1	0.9300	C22—C27	1.382 (6)
C2—C3	1.425 (5)	C22—C23	1.384 (5)
C2—H2	0.9300	C23—C24	1.383 (6)
C3—C4	1.335 (5)	C23—H23	0.9300
C3—C8	1.502 (5)	C24—C25	1.368 (7)
C4—C5	1.465 (5)	C24—H24	0.9300
C5—C6	1.539 (5)	C25—C26	1.364 (6)
C5—H5A	0.9700	C25—H25	0.9300
C5—H5B	0.9700	C26—C27	1.383 (5)
C6—C7	1.548 (4)	C26—H26	0.9300
C6—C13	1.562 (4)	C27—H27	0.9300
C6—H6	0.9800	C28—C29	1.483 (5)
C7—C10	1.515 (5)	C29—C34	1.372 (5)
C7—C8	1.554 (5)	C29—C30	1.383 (5)
C7—H7	0.9800	C30—C31	1.386 (6)
C8—C9	1.521 (6)	C30—H30	0.9300
C8—H8A	0.9800	C31—C32	1.366 (7)
C9—H9A	0.9600	C31—H31	0.9300
C9—H9B	0.9600	C32—C33	1.357 (6)
C9—H9C	0.9600	C32—H32	0.9300
C10—C11	1.522 (5)	C33—C34	1.393 (5)
C10—H10A	0.9800	C33—H33	0.9300
C11—C12	1.536 (4)	C34—H34	0.9300
C11—H11	0.9800	C35—H35A	0.9600
C12—C13	1.571 (5)	C35—H35B	0.9600
C12—C19	1.596 (5)	C35—H35C	0.9600
C13—C15	1.541 (4)		
			
C1—O1—C4	104.8 (3)	C13—C15—H15B	109.5
C20—O3—H3	109.5	H15A—C15—H15B	109.5
C21—O5—C11	116.5 (2)	C13—C15—H15C	109.5
C28—O7—C10	116.2 (3)	H15A—C15—H15C	109.5
C12—O8—H8	109.5	H15B—C15—H15C	109.5
C18—O9—H9	109.5	C17—C16—C13	112.6 (3)
C35—O10—H10	109.5	C17—C16—H16A	109.1
C2—C1—O1	111.5 (4)	C13—C16—H16A	109.1
C2—C1—H1	124.2	C17—C16—H16B	109.1
O1—C1—H1	124.2	C13—C16—H16B	109.1
C1—C2—C3	107.0 (4)	H16A—C16—H16B	107.8
C1—C2—H2	126.5	C18—C17—C16	110.6 (3)
C3—C2—H2	126.5	C18—C17—H17A	109.5
C4—C3—C2	105.9 (4)	C16—C17—H17A	109.5
C4—C3—C8	121.6 (3)	C18—C17—H17B	109.5
C2—C3—C8	132.4 (4)	C16—C17—H17B	109.5
C3—C4—O1	110.8 (3)	H17A—C17—H17B	108.1
C3—C4—C5	129.3 (3)	O9—C18—C17	111.9 (3)
O1—C4—C5	119.9 (3)	O9—C18—C19	109.9 (3)
C4—C5—C6	110.8 (3)	C17—C18—C19	116.2 (3)
C4—C5—H5A	109.5	O9—C18—H18	106.1
C6—C5—H5A	109.5	C17—C18—H18	106.1
C4—C5—H5B	109.5	C19—C18—H18	106.1
C6—C5—H5B	109.5	C20—C19—C14	103.4 (3)
H5A—C5—H5B	108.1	C20—C19—C18	113.7 (3)
C5—C6—C7	112.3 (3)	C14—C19—C18	106.8 (3)
C5—C6—C13	111.1 (3)	C20—C19—C12	115.3 (3)
C7—C6—C13	112.7 (2)	C14—C19—C12	109.2 (3)
C5—C6—H6	106.8	C18—C19—C12	108.0 (3)
C7—C6—H6	106.8	O2—C20—O3	121.3 (4)
C13—C6—H6	106.8	O2—C20—C19	122.5 (4)
C10—C7—C6	108.6 (3)	O3—C20—C19	116.1 (3)
C10—C7—C8	110.3 (3)	O4—C21—O5	123.9 (3)
C6—C7—C8	113.3 (3)	O4—C21—C22	123.8 (3)
C10—C7—H7	108.2	O5—C21—C22	112.2 (3)
C6—C7—H7	108.2	C27—C22—C23	120.0 (3)
C8—C7—H7	108.2	C27—C22—C21	122.3 (3)
C3—C8—C9	109.8 (3)	C23—C22—C21	117.5 (3)
C3—C8—C7	109.4 (3)	C24—C23—C22	119.2 (4)
C9—C8—C7	115.2 (3)	C24—C23—H23	120.4
C3—C8—H8A	107.4	C22—C23—H23	120.4
C9—C8—H8A	107.4	C25—C24—C23	120.6 (4)
C7—C8—H8A	107.4	C25—C24—H24	119.7
C8—C9—H9A	109.5	C23—C24—H24	119.7
C8—C9—H9B	109.5	C26—C25—C24	120.3 (4)
H9A—C9—H9B	109.5	C26—C25—H25	119.8
C8—C9—H9C	109.5	C24—C25—H25	119.8
H9A—C9—H9C	109.5	C25—C26—C27	120.2 (4)
H9B—C9—H9C	109.5	C25—C26—H26	119.9
O7—C10—C7	107.9 (3)	C27—C26—H26	119.9
O7—C10—C11	107.5 (2)	C22—C27—C26	119.7 (4)
C7—C10—C11	115.7 (3)	C22—C27—H27	120.1
O7—C10—H10A	108.5	C26—C27—H27	120.1
C7—C10—H10A	108.5	O6—C28—O7	123.2 (3)
C11—C10—H10A	108.5	O6—C28—C29	124.1 (4)
O5—C11—C10	109.5 (3)	O7—C28—C29	112.6 (3)
O5—C11—C12	111.7 (2)	C34—C29—C30	119.6 (3)
C10—C11—C12	109.4 (2)	C34—C29—C28	122.5 (3)
O5—C11—H11	108.7	C30—C29—C28	117.8 (4)
C10—C11—H11	108.7	C29—C30—C31	119.8 (4)
C12—C11—H11	108.7	C29—C30—H30	120.1
O8—C12—C11	100.7 (2)	C31—C30—H30	120.1
O8—C12—C13	109.3 (2)	C32—C31—C30	119.9 (4)
C11—C12—C13	111.3 (3)	C32—C31—H31	120.1
O8—C12—C19	104.7 (3)	C30—C31—H31	120.1
C11—C12—C19	114.1 (2)	C33—C32—C31	120.8 (4)
C13—C12—C19	115.4 (3)	C33—C32—H32	119.6
C15—C13—C16	107.8 (3)	C31—C32—H32	119.6
C15—C13—C6	110.1 (3)	C32—C33—C34	119.9 (4)
C16—C13—C6	109.1 (2)	C32—C33—H33	120.1
C15—C13—C12	113.1 (2)	C34—C33—H33	120.1
C16—C13—C12	108.5 (3)	C29—C34—C33	120.0 (4)
C6—C13—C12	108.1 (3)	C29—C34—H34	120.0
C19—C14—H14A	109.5	C33—C34—H34	120.0
C19—C14—H14B	109.5	O10—C35—H35A	109.5
H14A—C14—H14B	109.5	O10—C35—H35B	109.5
C19—C14—H14C	109.5	H35A—C35—H35B	109.5
H14A—C14—H14C	109.5	O10—C35—H35C	109.5
H14B—C14—H14C	109.5	H35A—C35—H35C	109.5
C13—C15—H15A	109.5	H35B—C35—H35C	109.5
			
C4—O1—C1—C2	−0.5 (5)	C11—C12—C13—C6	57.8 (3)
O1—C1—C2—C3	0.1 (5)	C19—C12—C13—C6	−170.1 (2)
C1—C2—C3—C4	0.4 (5)	C15—C13—C16—C17	−66.6 (4)
C1—C2—C3—C8	176.8 (4)	C6—C13—C16—C17	173.9 (3)
C2—C3—C4—O1	−0.8 (4)	C12—C13—C16—C17	56.3 (4)
C8—C3—C4—O1	−177.6 (3)	C13—C16—C17—C18	−58.8 (4)
C2—C3—C4—C5	−178.7 (4)	C16—C17—C18—O9	−176.5 (3)
C8—C3—C4—C5	4.4 (6)	C16—C17—C18—C19	56.3 (4)
C1—O1—C4—C3	0.8 (4)	O9—C18—C19—C20	−48.5 (4)
C1—O1—C4—C5	179.0 (4)	C17—C18—C19—C20	79.8 (4)
C3—C4—C5—C6	−11.2 (6)	O9—C18—C19—C14	64.9 (4)
O1—C4—C5—C6	171.0 (3)	C17—C18—C19—C14	−166.8 (3)
C4—C5—C6—C7	35.5 (4)	O9—C18—C19—C12	−177.7 (3)
C4—C5—C6—C13	162.8 (3)	C17—C18—C19—C12	−49.5 (4)
C5—C6—C7—C10	−178.8 (3)	O8—C12—C19—C20	159.2 (3)
C13—C6—C7—C10	54.8 (3)	C11—C12—C19—C20	50.1 (4)
C5—C6—C7—C8	−55.9 (4)	C13—C12—C19—C20	−80.7 (4)
C13—C6—C7—C8	177.8 (3)	O8—C12—C19—C14	43.3 (3)
C4—C3—C8—C9	105.7 (4)	C11—C12—C19—C14	−65.8 (3)
C2—C3—C8—C9	−70.2 (5)	C13—C12—C19—C14	163.4 (3)
C4—C3—C8—C7	−21.6 (5)	O8—C12—C19—C18	−72.5 (3)
C2—C3—C8—C7	162.5 (4)	C11—C12—C19—C18	178.4 (3)
C10—C7—C8—C3	168.7 (3)	C13—C12—C19—C18	47.6 (4)
C6—C7—C8—C3	46.7 (4)	C14—C19—C20—O2	36.5 (5)
C10—C7—C8—C9	44.4 (4)	C18—C19—C20—O2	151.9 (4)
C6—C7—C8—C9	−77.5 (4)	C12—C19—C20—O2	−82.6 (4)
C28—O7—C10—C7	−152.1 (3)	C14—C19—C20—O3	−140.7 (3)
C28—O7—C10—C11	82.5 (3)	C18—C19—C20—O3	−25.3 (5)
C6—C7—C10—O7	−174.8 (2)	C12—C19—C20—O3	100.1 (4)
C8—C7—C10—O7	60.5 (3)	C11—O5—C21—O4	0.7 (5)
C6—C7—C10—C11	−54.4 (4)	C11—O5—C21—C22	−179.3 (3)
C8—C7—C10—C11	−179.2 (3)	O4—C21—C22—C27	−150.8 (4)
C21—O5—C11—C10	−97.8 (3)	O5—C21—C22—C27	29.2 (5)
C21—O5—C11—C12	140.8 (3)	O4—C21—C22—C23	24.9 (5)
O7—C10—C11—O5	53.9 (3)	O5—C21—C22—C23	−155.1 (3)
C7—C10—C11—O5	−66.7 (3)	C27—C22—C23—C24	0.1 (6)
O7—C10—C11—C12	176.6 (2)	C21—C22—C23—C24	−175.7 (3)
C7—C10—C11—C12	56.0 (4)	C22—C23—C24—C25	1.9 (6)
O5—C11—C12—O8	−179.3 (2)	C23—C24—C25—C26	−2.0 (7)
C10—C11—C12—O8	59.3 (3)	C24—C25—C26—C27	−0.1 (6)
O5—C11—C12—C13	65.0 (3)	C23—C22—C27—C26	−2.1 (6)
C10—C11—C12—C13	−56.4 (3)	C21—C22—C27—C26	173.4 (3)
O5—C11—C12—C19	−67.7 (3)	C25—C26—C27—C22	2.1 (6)
C10—C11—C12—C19	170.8 (3)	C10—O7—C28—O6	9.3 (5)
C5—C6—C13—C15	−60.4 (3)	C10—O7—C28—C29	−168.8 (3)
C7—C6—C13—C15	66.6 (3)	O6—C28—C29—C34	−176.1 (4)
C5—C6—C13—C16	57.7 (4)	O7—C28—C29—C34	1.9 (5)
C7—C6—C13—C16	−175.3 (3)	O6—C28—C29—C30	2.3 (6)
C5—C6—C13—C12	175.5 (3)	O7—C28—C29—C30	−179.6 (3)
C7—C6—C13—C12	−57.4 (3)	C34—C29—C30—C31	0.7 (6)
O8—C12—C13—C15	−174.7 (3)	C28—C29—C30—C31	−177.7 (4)
C11—C12—C13—C15	−64.4 (4)	C29—C30—C31—C32	0.3 (7)
C19—C12—C13—C15	67.7 (4)	C30—C31—C32—C33	−1.2 (8)
O8—C12—C13—C16	65.8 (3)	C31—C32—C33—C34	1.1 (7)
C11—C12—C13—C16	176.0 (2)	C30—C29—C34—C33	−0.8 (6)
C19—C12—C13—C16	−51.9 (3)	C28—C29—C34—C33	177.6 (3)
O8—C12—C13—C6	−52.5 (3)	C32—C33—C34—C29	−0.1 (6)

### Hydrogen-bond geometry (Å, º)

**Table:**

*D*—H···*A*	*D*—H	H···*A*	*D*···*A*	*D*—H···*A*
O3—H3···O10	0.84	1.79	2.626 (5)	177
O8—H8···O2^i^	0.84	2.03	2.728 (3)	141
O10—H10···O8^ii^	0.84	2.26	2.967 (4)	142
C15—H15*A*···O3	0.96	2.35	3.231 (5)	152

Symmetry codes: (i) −*x*, *y*+1/2, −*z*+1/2; (ii) −*x*, *y*−1/2, −*z*+1/2.
