# Supplementary figures and images for: Crystal structure of (3S*,4S*,4aS*,5R*,6R*,6aS*,7R*,11aS*,11bR*)-5,6-bis(benzo­yloxy)-3,4a-dihy­droxy-4,7,11b-trimethyl-1,2,3,4,4a,5,6,6a,7,11,11a,11b-dodeca­hydro­phenanthro[3,2-b]furan-4-carb­oxy­lic acid methanol monosolvate
Source: Acta Crystallogr E Crystallogr Commun. 2015 Sep 12;71(Pt 10):o739–40. doi: 10.1107/S2056989015016461 (PMC4647408; doi:10.1107/S2056989015016461)

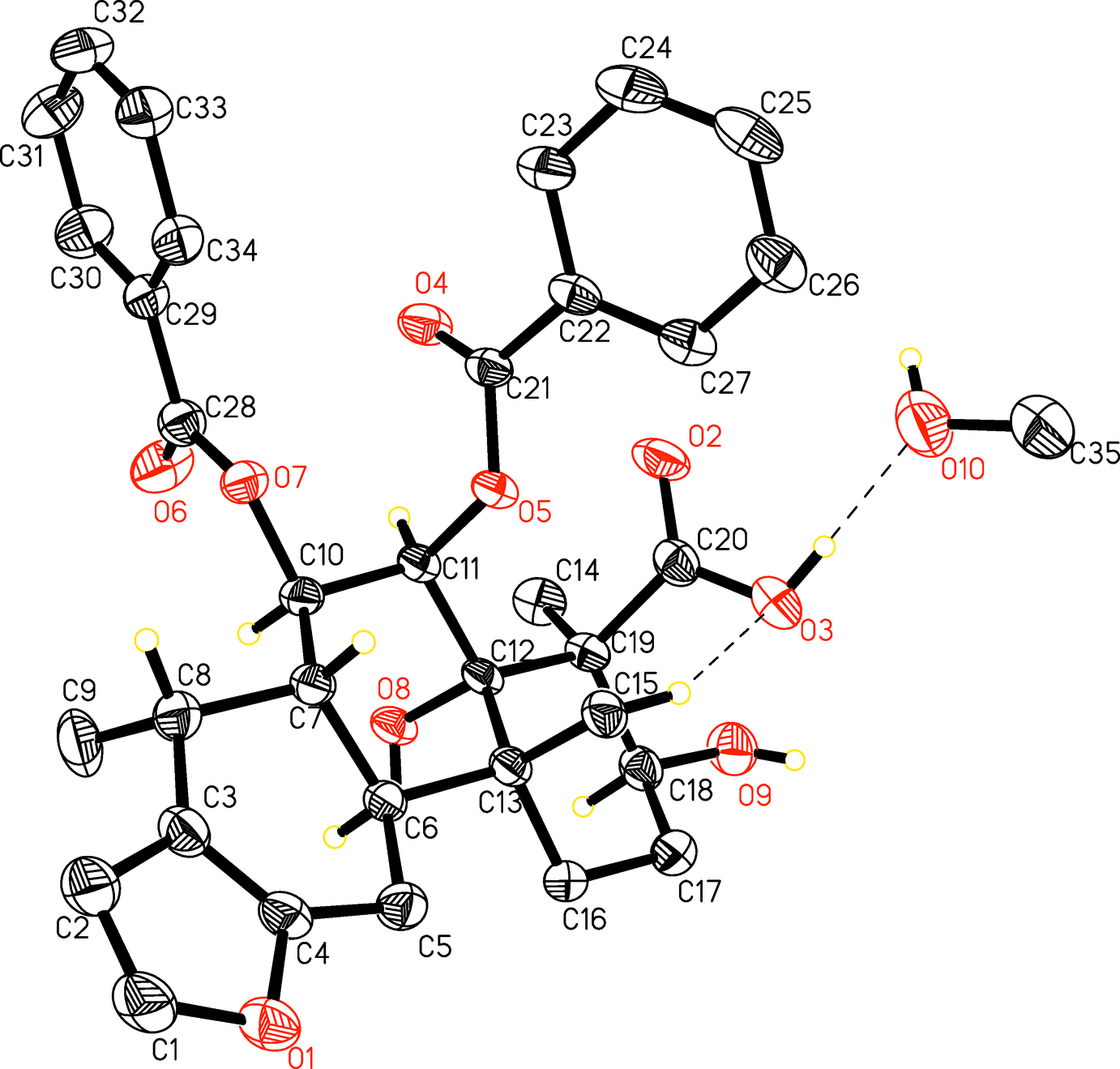

Supplement: Supplementary file 4 [file e-71-0o739-fig1.tif]

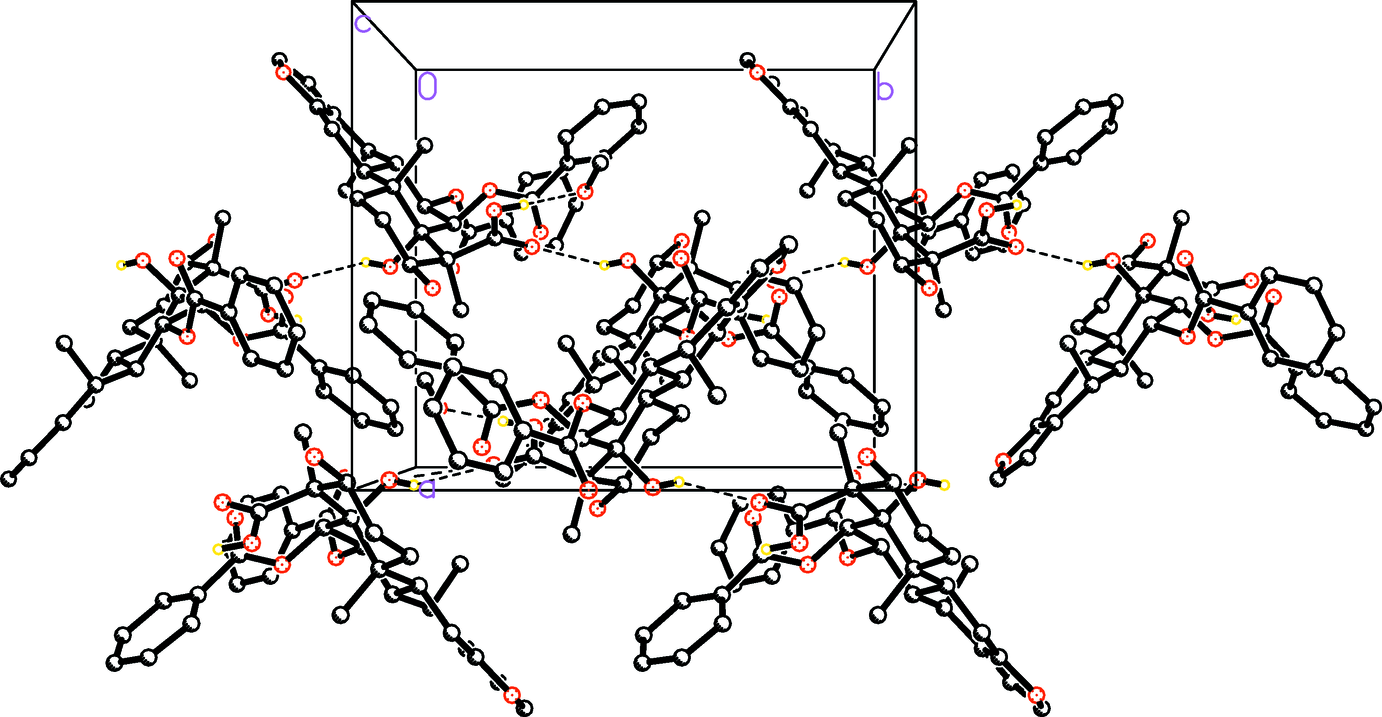

Supplement: Supplementary file 5 [file e-71-0o739-fig2.tif]
